# Modified Atkins Diet therapy during pregnancy for refractory idiopathic generalized epilepsy

**DOI:** 10.1016/j.ebr.2025.100832

**Published:** 2025-10-06

**Authors:** Ryoichi Inoue, Elizabeth Weinandy, Sarita Maturu, Nabil Khandker

**Affiliations:** a Department of Neurology, The Ohio State University Wexner Medical Center, 395 W 12th Ave #7, Columbus, OH 43210, United States; b Department of Nutrition and Dietetics, The Ohio State University Wexner Medical Center, 453 W 10th Ave, Columbus, OH 43210, United States; c Department of Neurology, Epilepsy Division, The Ohio State University Wexner Medical Center, 395 W 12th Ave #7, Columbus, OH 43210, United States

**Keywords:** Diet therapy, Modified Atkins Diet, Idiopathic generalized epilepsy, Fetal development, Maternal nutrition, Antiseizure medication, Oligohydramnios

## Abstract

The management of refractory epilepsy in pregnancy is challenging, and diet therapy has been rarely reported. We report a case of a 25-year-old woman with refractory idiopathic generalized epilepsy who achieved prolonged seizure freedom with the Modified Atkins Diet (MAD) during pregnancy. Her epilepsy began at age 3, with seizures occurring every two months despite multiple antiseizure medications and vagus nerve stimulation. Three months before her first pregnancy, MAD was initiated with a carbohydrate limit of 30 g daily, resulting in immediate seizure freedom. During the pregnancy, carbohydrate intake was gradually increased to 60–70 g daily to support fetal growth. She remained seizure-free for 10 months before experiencing breakthrough seizures in the third trimester. Oligohydramnios was subsequently detected, and she underwent induction of labor with vaginal delivery at 37 weeks. After delivery, she discontinued MAD for breastfeeding and remained seizure-free for 13 months. In her second pregnancy without MAD, she experienced two breakthrough seizures in the second trimester. She later achieved 23 months of seizure freedom. Both children have met developmental milestones appropriately. This case adds to the limited literature on diet therapy in pregnancy and is, to our knowledge, the first involving generalized epilepsy. The case suggests MAD may provide seizure control during pregnancy, even with possible sustained benefit after discontinuation. However, given the occurrence of oligohydramnios and limited safety data, larger studies are needed to clarify the efficacy, safety, and long-term outcomes of diet therapy during pregnancy.

## Highlights

• MAD reduced seizure frequency in pregnancy with generalized epilepsy.
• Sustained seizure control was observed even after MAD discontinuation.
• MAD was liberalized to support fetal growth during pregnancy.
• Oligohydramnios occurred during pregnancy on MAD.

## 1 Introduction

Drug-resistant epilepsy affects a subset of individuals with epilepsy, including those who become pregnant. This highlights the need for potential non-pharmacological therapies that can be safely used during pregnancy, such as diet therapy. The ketogenic diet is a structured, highly restrictive therapy requiring a 4:1 ratio of fat to protein plus carbohydrates. In contrast, the Modified Atkins Diet (MAD) is more flexible, allowing generous intake of protein and fat, and typically follows a 1:1 ratio and limits carbohydrate intake to around 20 g daily [1]. While diet therapy is an effective adjunctive treatment for refractory epilepsy [1], its use in pregnancy has rarely been reported [2,3]. A recent international consensus statement provides specific recommendations on diet therapy during pregnancy [4]. In that survey, 60 % of experts did not recommend using ketogenic diet therapies (KDTs) during pregnancy due to concerns regarding fetal health and a lack of information. Similarly, a global practice survey reported that 47 % of clinicians considered pregnancy an absolute contraindication and 42 % a relative contraindication to initiating KDTs [5]. Therefore, more data is needed. Here, we present a case of a patient with refractory idiopathic generalized epilepsy who was maintained on MAD throughout pregnancy.

## 2 Case presentation

A 25-year-old female with idiopathic generalized epilepsy presented at the epilepsy clinic. Her seizures began at age 3, characterized by absence seizures and generalized tonic-clonic seizures. Vagus nerve stimulation (VNS) was placed at the age of 10, resulting in the resolution of absence seizures. VNS settings included an output current of 2.00 mA, signal frequency of 25 Hz, pulse width of 250 µs, and a 10 % duty cycle. Despite treatment with antiseizure medications (ASMs) including levetiracetam (LEV) 1750 mg in the morning and 1750 mg in the evening (1750 mg/1750 mg), lamotrigine (LTG) 250 mg/250 mg, ethosuximide (ESM) 500 mg/500 mg, and clobazam (CLB) 5 mg/10 mg, she continued to have breakthrough seizures every 6 to 12 months. All breakthrough events were generalized tonic–clonic seizures. Her electroencephalography showed independent right and left frontal spike discharges and generalized polyspike and wave discharges. Her MRI revealed no underlying epileptogenic lesion.

**Fig. 1:**
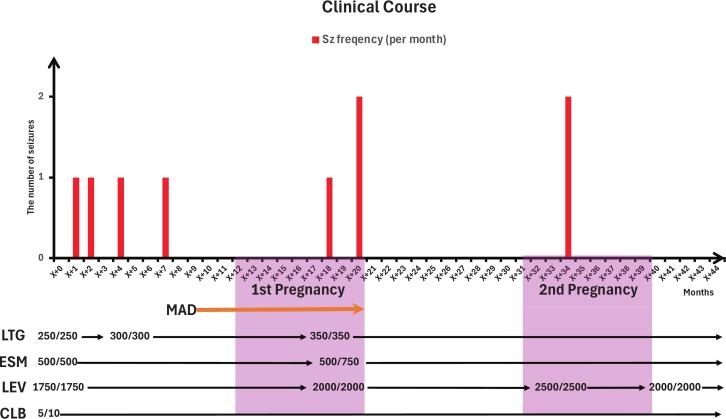
Clinical course of seizure frequency, diet therapy, and antiseizure medication (ASM) adjustments. The horizontal axis represents time in months, beginning with the patient's initial presentation to the epilepsy clinic (denoted as X+0). The vertical axis shows the number of seizures per month. Red bars indicate monthly seizure frequency. ASM dosages are depicted below the graph along the timeline, abbreviated in milligrams (mg) and expressed as "dose/dose" for morning and evening administrations, with black arrows indicating periods of stable dosing. The Modified Atkins Diet (MAD) was initiated prior to conception and continued into pregnancy, as shown by the yellow arrow. Each pink-shaded area represents the period of pregnancy, aligned with the timeline. Breakthrough seizures and ASM dose adjustments are reflected at corresponding time points.

One year prior to her pregnancy, she had an increase in seizure frequency to approximately two seizures per month over the next eight months (Fig. 1). The patient and her husband were concerned about her safety and requested diet therapy primarily for seizure reduction. She was also interested in the ancillary potential for weight loss. At her request, we consulted a dietitian and initiated MAD with a carbohydrate intake of 30 g per day as adjunctive therapy. She achieved immediate seizure freedom. Three months later, she had a planned pregnancy. Despite counseling about the current recommendations against diet therapy during pregnancy, she opted to continue MAD.

To ensure adequate nutrition for the fetus, we gradually increased carbohydrate intake, primarily to meet specific micronutrient needs from non-starchy vegetables and foods naturally higher in carbohydrates, such as fruits and dairy. This was particularly important in the third trimester when fetal growth is most rapid. Therefore, carbohydrate intake was increased from about 30 to 45–50 g, and later to 60–70 g daily (Table 1). Protein and fat intake remained stable. Routine ketone monitoring was not performed during MAD because we were not targeting a specific level of ketosis. Additionally, she continued a daily prenatal vitamin and folic acid, and later initiated iron and vitamin D supplementation for vitamin D insufficiency, consistent with pregnancy recommendations [6,7].

**Table 1:** Overview of the patient's clinical parameters, antiseizure medication (ASM) adjustments, serum ASM levels, and seizure frequency across two pregnancies and through the most recent follow-up. ASM abbreviations: LEV – levetiracetam, LTG – lamotrigine, ESM – ethosuximide, CLB – clobazam.

Month	X + 9	X + 13	X + 14	X + 16	X + 19	X + 20	X + 30	X + 34	X + 38	X + 39	X + 41	X + 50	X + 56	X + 69	X + 80
Pregnancy Weeks	Prior to 1st pregnancy- Baseline	6 weeks	10 weeks	20 weeks	28 weeks	36 weeks	Prior to 2nd pregnancy	14 weeks	32 weeks	37 weeks	1.5 months after delivery	1 year after 2nd pregnancy			
Weight (kg)	78.02	NA	55.75	58.97	67.59	69.85	50.3	58.1	68.5	70.8	58.9	50.2	49.6	50.3	50.6
BMI (kg/m2)	32.5	NA	23.2	24.6	28.2	29.1	21	24.2	28.53	29.5	24.5	20.9	20.7	20.9	21.1
MAD duration (months)	0	5	6	8	11	12									
Carb (g/day)	30	50	45–60	45–60	60	60–70									
Protein (g/day)	90–110	NA	NA	NA	NA	NA									
Fat (g/day)	110–130	NA	NA	NA	NA	NA									
Other supplements(PNV: prenatal vitamin, FA: folic acid)	PNV, FA 4 mg/day	PNV, FA 4 mg/day	PNV, FA 4 mg/day	PNV, FA 4 mg/day, vitamin D 2000 units, ferrous sulfate 60 mg	PNV, FA 4 mg/day, vitamin D 2000 units, ferrous sulfate 60 mg	PNV, FA 4 mg/day, vitamin D 2000 units, ferrous sulfate 60 mg	PNV, FA 4 mg/day, vitamin D 2000units	PNV, FA 4 mg/day, vitamin D 2000units	PNV, FA 4 mg/day, vitamin D 2000units	PNV, FA 4 mg/day, vitamin D 2000units	PNV, vitamin D 2000 units	PNV, vitamin D 2000 units	PNV, vitamin D 2000 units	PNV, vitamin D 2000 units	PNV, vitamin D 2000 units
Cholesterol (mg/dl)	212			258					306				264		260
Triglycerides (mg/dl)	85			134					356				50		62
HDL (mg/dl)	80			104					94				102		119
LDL (mg/dl)	115			127					141				152		129
Non-HDL Cholesterol (mg/dl)	132			154					212				162		141
LTG level (mg/ml)		3.6		5.1	4.9	5.5		4	3.8		9.8				8.4
LTG dose (mg, morning/evening)	300/300	300/300	300/300	300/300	increased to 350/350	350/350	350/350	350/350	350/350	350/350	350/350	350/350	350/350	350/350	350/350
LEV level (mcg/ml)		23.5		41	43.7	46.7		18.9	25.2		47.6				41.2
LEV dose(mg, morning/evening)	1750 /1750	1750 /1750	1750 /1750	1750 /1750	Increased to 2000/2000	2000/2000	2000/2000	Increased to 2500/2500	2500/2500	2500/2500	Reduced to 2000/2000 (pre-pregnancy dose)	2000/2000	2000/2000	2000/2000	2000/2000
ESM level (mcg/ml)		NA		45	57	42		46	34		60				70
ESM dose(mg, morning/evening)	500/500	500/500	500/500	500/500	Increased to 500/750	500/750	500/750	500/750	500/750	500/750	500/750	Reduced to 500/500	Increased to 500/750	500/750	500/750
Seizures per month	0.5	0	0	0	1	2	0	2	0	0	0	0	1	0	0
Sz free interval (months)	1	6	7	9	0	1	9	13	3	4	6	15	22	12	23

She maintained 10 months of seizure freedom until a breakthrough seizure occurred at 28 weeks' gestation despite therapeutic ASM levels. This led to an increase in LTG to 350 mg/350 mg, followed by LEV to 2000 mg/2000 mg and ESM to 500 mg/750 mg. At 36 weeks of pregnancy, she had two breakthrough seizures, likely triggered by poor sleep and a significant reduction in ESM level while other ASM levels remained therapeutic. No changes were made to VNS settings. The following day, oligohydramnios (amniotic fluid index 2.29 cm) was detected, and she was admitted for induction. She delivered vaginally at 37 weeks and 1 day. The first child was born at a weight of 2370 g (12 %ile; Z-score = –1.16; Fenton 2013, girls, 22–50 weeks).

After delivery, she discontinued MAD for breastfeeding and partially because of adherence fatigue. However, she remained seizure-free for 13 months. She later became pregnant again without resuming MAD. She experienced two breakthrough seizures in the second trimester with a significant reduction in LEV levels, though still within therapeutic range, resulting in an increase in LEV to 2500 mg/2500 mg. She delivered at 38 weeks. The second child was born at a weight of 3040 g (49 %ile; Z-score = –0.02; Fenton 2013, girls, 22–50 weeks).

ASM doses were later decreased to LEV 2000 mg/2000 mg and ESM 500 mg/500 mg. She remained seizure-free for 22 months, followed by a breakthrough seizure when ASM levels were not checked. As of her last follow-up, she maintained 23 months of seizure freedom.

Both children have demonstrated normal developmental milestones, with no epilepsy or congenital malformations. Growth parameters remained on the very small side, consistent with parental stature, but both children grew steadily along their respective curves. At age 5, the first child weighed 12.9 kg (<1%ile; Z-score = –3.10) with height 0.972 m (<1%ile; Z-score = –2.54). At age 3, the second child weighed 10.5 kg (<1%ile; Z-score = –2.78) with height 0.826 m (<1%ile; Z-score = –3.10). Follow-up percentiles/Z-scores used CDC growth charts (girls, 2–20 years).

## 3 Discussion

We report a case of refractory epilepsy maintained on MAD as an adjunct therapy throughout pregnancy. Significant seizure reduction was achieved following MAD initiation, although the patient developed oligohydramnios.

To our knowledge, only a few reports have described dietary therapy during pregnancy in epilepsy. Van der Louw et al described two cases of focal epilepsy: one treated with classic KD as monotherapy and the other managed with MAD as adjunctive therapy [2]. While previous reports involved patients with focal epilepsy, this case highlights that MAD was also effective in a pregnant patient with generalized epilepsy. Interestingly, our patient maintained prolonged seizure control even after discontinuation of MAD, including throughout most of her second pregnancy, with breakthrough seizures only suspected to be secondary to reduction in LEV levels, and continuing until her most recent follow-up. While this may be influenced by potential confounding factors such as ASM optimization, as well as the modulatory effects of pregnancy and breastfeeding on seizure control, it also raises the possibility of a prolonged effect of MAD, as suggested in prior studies where seizure reduction persisted after diet discontinuation [8]. Furthermore, most breakthrough seizures after starting MAD occurred alongside significant reductions in ASM levels, further supporting this possibility. For patients with intractable epilepsy or on ASMs with a greater risk of major congenital malformations, diet therapy may offer a non-pharmacological option for seizure reduction.

In our case, the patient developed oligohydramnios during her first pregnancy. Whether this complication was related to MAD or other factors including the use of ASMs remains uncertain. However, restriction of nutrient delivery inherent to MAD is a plausible contributor, particularly given that no obstetric complications were observed in the second pregnancy despite ASM adjustments. Van der Louw et al reported bilateral ear deformities of unknown significance in an offspring exposed to MAD in utero, while no complications were seen in another offspring exposed to KD [2]. Kramer and Smith also reported a patient with glucose transporter type 1 deficiency (Glut1DS) who continued ketogenic therapy throughout pregnancy; the infant, later diagnosed with Glut1DS, was started on ketogenic diet and showed normal development [3]. Moreover, animal studies have shown potential risks, including altered embryonic organ growth and neurodevelopmental changes [9]. Thus, current expert consensus statements and surveys recommend against the use of diet therapy during pregnancy [4,5].

Additionally, it is important to note that MAD was liberalized in our case to support fetal growth, with carbohydrate intake increased beyond a traditional MAD approach, mainly through fruits and non-starchy vegetables. We also encouraged the patient to incorporate new, nutritious foods in more palatable forms, such as spinach smoothies.

## 4 Conclusion

In summary, MAD may offer seizure reduction as a non-pharmacological therapy during pregnancy, with the potential for prolonged benefit after discontinuation. However, its safety remains uncertain due to the occurrence of oligohydramnios in our case and the limited number of reported cases. Larger studies are required to further evaluate the efficacy, risks, and long-term outcomes of MAD during pregnancy.

## Ethical statement

Institutional review board approval was not required for a single-patient case report, as per our institution's policy. Written informed consent was obtained from the patient for publication of this case report. The patient reviewed and approved the final version of the manuscript.

## Funding sources

This research did not receive any specific grant from funding agencies in the public, commercial, or not-for-profit sectors.

## CRediT authorship contribution statement

**Ryoichi Inoue:** Writing – review & editing, Writing – original draft, Visualization, Methodology, Conceptualization. **Elizabeth Weinandy:** Writing – review & editing, Supervision. **Sarita Maturu:** Writing – review & editing, Supervision. **Nabil Khandker:** Writing – review & editing, Supervision.

## Declaration of competing interest

The authors declare that they have no known competing financial interests or personal relationships that could have appeared to influence the work reported in this paper.
